# Preparation and Application of an Innovative Thrombocyte/Leukocyte-Enriched Plasma to Promote Tissue Repair in Chelonians

**DOI:** 10.1371/journal.pone.0122595

**Published:** 2015-04-22

**Authors:** Francesco Di Ianni, Elisa Merli, Francesca Burtini, Virna Conti, Igor Pelizzone, Rosanna Di Lecce, Enrico Parmigiani, Gian Paolo Squassino, Maurizio Del Bue, Enrico Lucarelli, Roberto Ramoni, Stefano Grolli

**Affiliations:** 1 Department of Veterinary Science, Università degli Studi di Parma, Via Del Taglio 10, 43126, Parma, Italy; 2 Clinica Veterinaria Ambulatorio Squassino, Asti, Italy; 3 Osteoarticular Regeneration Laboratory, Rizzoli Orthopaedic Institute, 40136, Bologna, Italy; University of Bari, ITALY

## Abstract

Platelet concentrates are widely used in mammalian regenerative medicine to improve tissue healing. Chelonians (Testudines) would benefit from the application of thrombocyte preparations to regenerate damaged tissues, since traumatic injuries are leading causes of morbidity and mortality for both wild-living and domesticated animals. The aim of this study was to establish a protocol that optimized the recovery of the thrombocytes from blood samples and to show the efficacy of thrombocyte-enriched plasma in chelonians. Peripheral blood samples were obtained from *Testudo spp*. (n = 12) and *Trachemys scripta elegans* (n = 10). Blood cells were fractionated by sodium diatrizoate-sodium polysucrose density gradient using a two-step centrifugation protocol. Thrombocytes and leukocytes were isolated and resuspended to obtain thrombocyte-leucocyte rich plasma (TLRP). The mean recovery of leukocytes and thrombocytes was 48.9% (±4.0 SEM, n = 22) of the whole blood cell content. No statistically significant difference was observed between blood samples collected from different turtle species. The ability of TLRP to form a gel was evaluated by adding variable concentrations of calcium gluconate at room temperature and at 37°C. A reliable and consistent clotting of the TLRP was obtained in glass tubes and dishes by adding 5-20% v/v of a 100 mg/ml solution of calcium gluconate. Furthermore, in order to test the clinical efficacy of TLRP, a preliminary evaluation was performed on four turtles (Testudo spp.) with traumatic injuries. In all the four animals, a successful clinical outcome was observed. The results demonstrated that a thrombocyte-enriched plasma, comparable to mammalian platelet rich plasma, can be prepared from chelonian blood samples. Furthermore, although the low number of cases presented does not allow definitive conclusions from a clinical point of view, their outcome suggests that TLRP application could be further investigated to improve the healing process of both soft and hard tissue injuries in chelonians.

## Introduction

Chelonians have become increasingly popular pets in recent years and internal medicine and surgery of turtles currently involve a growing number of veterinary practitioners. Tortoises (*Testudo spp*.) and red-eared sliders (*Trachemys scripta elegans*) are the most bred species of turtles in Europe. Tortoises are included in appendix ii of the Convention on International Trade of Endangered Species (C.I.T.E.S., Washington D.C. USA, 1973).

Traumatic injuries are one of the most common problems which veterinary practitioners must face, for turtles living in the wild and in captivity. Trauma is due to a variety of causes, including being attacked by predators (birds of prey, wolves, dogs, etc.), run over by a car, hit by a lawn mower, falling from heights, etc. Even though limb, head and different soft tissue injuries can be severely debilitating, carapace fractures are particularly frustrating. Indeed, they may cause long-lasting disability and sufference, since they usually require a long time to heal and to obtain good anatomical and functional recovery [1–3]. Along with the slow regeneration of hard tissue, wound healing in turtles can be impaired or weakened by the ease of tissue contamination by bacteria and/or parasites, and by the difficulty to isolate and protect the damaged area. A growing number of therapeutic strategies have been applied to bypass these problems, but to date none of them can be considered completely satisfactory. Indeed, current treatment for fracture stabilization based on the use of non-organic materials such as screws, epoxy resins, pins, and/or wires, can induce adverse reactions which delay the healing process [1, 4].

In recent years, regenerative medicine has gained a strong interest within the research community, leading to the development of innovative cellular and molecular strategies aimed at stimulating soft and hard tissue healing in vivo. Wound healing is a complex biological process that requires the synergistic interaction between different factors to ensure both anatomical and functional tissue recovery. Haemostasis, cell proliferation and differentiation, and tissue remodelling are the key processes driving wound healing. Their success requires the fine tuning of coordinated cell-to-cell interactions coupled with appropriate soluble growth factor-mediated signalling [5, 6]. Platelets are key players of this process. These cells, indeed, synthesize, store and release upon activation, several growth factors (including Epidermal Growth Factor, Basic Fibroblast Growth Factor, Insulin Like Growth Factor I and II, Vascular Endothelial Growth Factor) and chemotactic molecules involved in haemostasis and cell proliferation [7]. Growth factors released by platelets can stimulate proliferation of mesenchymal cells, fibroblasts, osteoblasts, endothelial cells [8, 9], and also modulate migration and activation of macrophages, monocytes and polymorphonucleated cells.

The discovery of the pivotal role played by platelets in tissue recovery has supported the development of the so-called ‘platelet concentrates’, a group of blood component characterized by an elevated platelet concentration [10]. Among these, Platelet Rich Plasma (PRP) and Platelet Gel (PG) have been applied in a large variety of clinical applications on the premise that they can actively contribute to the healing process by locally delivering a high concentration of growth factors. PRP and PG have been used to enhance tissue recovery in orthopaedics, periodontal surgery, plastic surgery and soft tissue surgery both in human and veterinary medicine. [11–13]. The clinical application of platelet concentrates in veterinary medicine has mainly been focused on a restricted number of domestic mammal species, such as horses, cats and dogs [14–16]. Interestingly, to the authors’ knowledge, there is currently no report concerning thrombocyte-based preparations derived from reptile blood. A protocol for the preparation of platelet concentrates starting from chelonian whole blood would be of interest, in particular for the treatment of traumatic injuries of the carapace, limbs and exposed soft tissues. Although several therapeutic approaches are available for the treatment of traumatic wounds in turtles, the use of a simple blood derivative capable of sealing wounds and stimulating their healing would be extremely helpful.

In the present work we describe a protocol for the preparation of plasma enriched in thrombocytes and leukocytes from four different turtle species, *T*. *hermanni*, *T*. *graeca*, *T*. *marginata* and *T*. *scripta elegans*, starting from peripheral blood, by using a density gradient centrifugation procedure. Furthermore, we describe the preparation of thrombocyte-enriched plasma gel, starting from both freshly prepared thrombocyte concentrate and frozen/thawed thrombocyte lysate. Finally, we describe the clinical outcome of the application of the thrombocyte-enriched plasma in four different cases of traumatic injuries in chelonians.

## Materials and Methods

All the materials used in the present study were purchased from Sigma Aldrich (Milan, Italy) unless otherwise stated.

### Specimen collection

Blood samples were collected either from the caudal or cervical vein of *Testudo spp*. (n = 12) and *Trachemys scripta* (n = 10) using sterile syringes containing Acid Citrate Dextrose (ACD) solution (sodium citrate, citric acid, dextrose) as an anticoagulant. Needle caliber was 25 G for animals weighing less than 500 g, 23 G for animals weighing between 500 g and 2 kg, and 22 G for larger animals. A few microliters of sterile ACD were aspirated into the needle before drawing to prevent blood clotting. Collected sample volumes ranged between 0.8 and 3.2 ml, depending on the animal’s weight. The blood samples were processed immediately or stored at room temperature for a maximum of 6 hours. Heparin, most commonly used as anticoagulant in reptiles, was not utilized since it inhibits plasma calcium-driven coagulation needed for the preparation of the plasma gel (see below). An informed consent was obtained from the owners of the animals prior to study enrollment. Procedures were approved by the local Ethic Committee of the University of Parma.

Total red blood cell, white blood cell and thrombocyte counts were performed according to Natt and Herrick (N&H) [17] using a Burker camera. Since N&H staining does not allow a reliable differentiation between leukocytes (LL) and thrombocytes (TT) [18], and considering that our goal was to prepare a plasma fraction enriched in leukocytes and thrombocytes (LL/TT), the total LL and TT count was performed and reported. Briefly for cell count, each blood sample was diluted 1:100 in N&H solution and cells were counted after 5 minutes, at 400x total magnification in a Burker camera.

### Preparation of thrombocyte and leukocyte rich plasma

**Fig 1:**
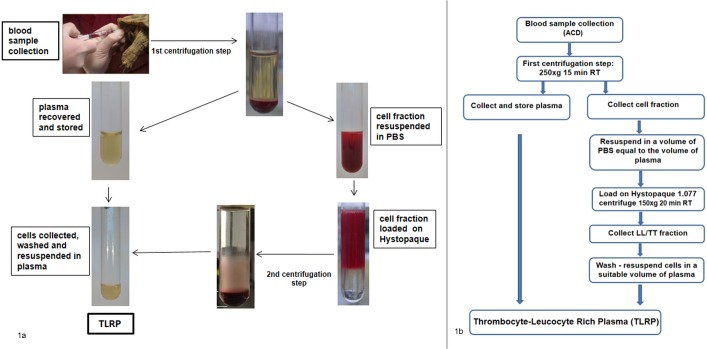
TLRP preparation from turtle blood samples. Schematic representation (a) and flowchart (b) of the preparation of the Thrombocytes and Leukocytes Rich Plasma (TLRP) from blood sample collected from the caudal or cervical vein of *Testudo spp* and *T*. *scripta elegans*. After a first centrifugation step, blood cells were fractionated on a Histopaque 1.077 gradient and leukocytes/thrombocytes (LL/TT) collected to prepare the TLRP. A step by step summary is available as Supporting information, S5 Fig.

**Fig 2:**
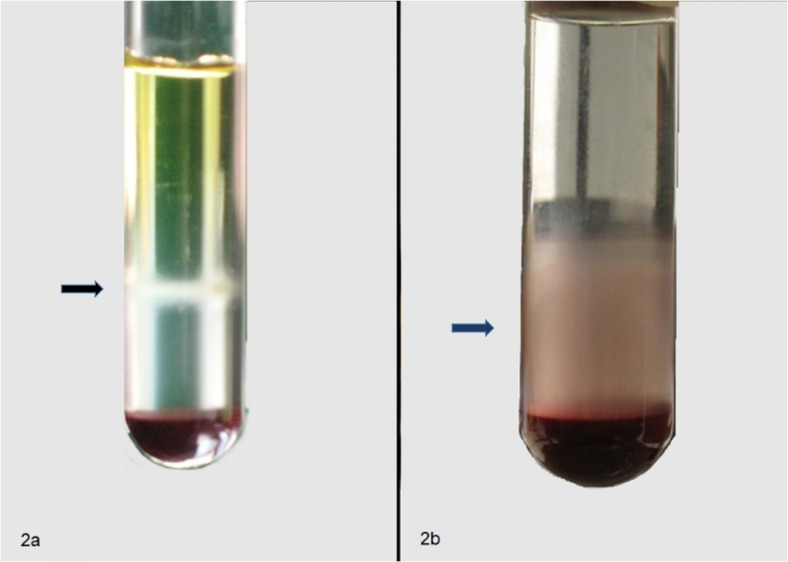
Blood cells fractionation on Histopaque 1.077 performed applying (a) 480g, 30 min or (b) 150g, 20 min centrifugation step, RT. Cell counting demonstrated a higher recovery of leukocytes and thrombocytes with method (b).

Separation of leukocytes and thrombocytes was performed modifying the method proposed by Morgan et al. [19] that describes a density fractionation protocol using a solution of sodium diatrizoate polysucrose (density 1.077 g/ml), commercially available as Histopaque 1.077. Each blood sample was first centrifuged at 250xg for 15 minutes in a swing-out rotor centrifuge (ALC model 4233R, Milan, Italy), to separate cell-poor plasma from the blood-cell fraction (Fig 1). The cell-poor plasma fraction was then collected, quantified and stored at either 4°C or at room temperature in a sterile tube, until it was used for LL/TT rich plasma preparation (see below). The blood-cell fraction was diluted with a volume of sterile Phosphate Buffered Saline (PBS) pH 7.2, equal to that of the collected cell-poor plasma, and layered on Histopaque 1.077 pre-warmed at room temperature. The volume ratio between the resuspended blood cell fraction and Histopaque was 1:1. Cells were then centrifuged for 20 minutes at 150xg. After centrifugation, the enriched LL/TT cell fraction, consisting of an opaque ring and a diffused cloud above the Histopaque layer (Fig 2B), was collected. Cells were washed twice in 4 ml of sterile PBS at 250 x g for 10 minutes, resuspended in a small volume of cell-poor plasma (100–150 μl) and then counted by the N&H method. Leucocyte and thrombocyte recovery was calculated as the percentage of the total number of LL/TT cells (LL/TT_t_) present in the blood sample compared to those recovered (LL/TT_r_) after processing, i.e. % of cell recovery = (100 x LL/TT_r_)/LL/TT_t_. After cell counting, LL/TT fraction was diluted in an appropriate volume of sterile cell-poor plasma to prepare a LL/TT rich plasma with a 3–4 fold-increased cell content compared to the initial blood sample. This LL/TT enriched cell fraction has been termed ‘thrombocyte-leucocyte rich plasma’ (TLRP).

### Preparation of TLRP gel

Different conditions were evaluated to obtain reliable and reproducible thrombocyte activation and plasma gelation *in vitro*. The average clotting time was determined at room temperature (RT, 22–24 °C) and at 37 °C, in different plate and tube substrates (i.e. plastic and glass), by adding a stock solution of 100 mg/ml of calcium gluconate at a final concentration ranging from 2.5 to 20% v/v to 200 μl of either pooled cell-poor plasma or TLRP. Each pool was obtained by mixing samples prepared from three different animals. Clotting was evaluated every 2 minutes by visual inspection starting both from freshly prepared TLRP and from thawed cell lysate. Cell lysate was prepared by freezing TLRP at -20°C and then thawing it at 37 °C.

### Cell staining

Cell smears were prepared from whole blood and TLRP samples. Cells were stained with Diff Quick (Bio-Optica, Milano, Italy), May Grunwald—Giemsa (Bio-Optica, Milano, Italy) and Periodic Acid Schiff (PAS) (Bio-Optica, Milano, Italy), according to manufacturers’ instruction. Slides were examined by light microscopy (400x or 1000x total magnification) with a Nikon Eclipse E800 microscope (Nikon Instruments, Firenze, Italy).

### Application of TLRP: case histories

Four turtles (Testudo spp.) with traumatic injuries were treated with TLRP. After initial stabilization, all turtles were hospitalized in an environment with controlled temperature (28 °C in the day and 24 °C during the night) and humidity (70%) and antibiotic therapy was started. A brief description of the four clinical cases is reported in Table 1, while a more accurate description is available as Supporting Information (S1 Fig, S2 Fig, S3 Fig and S4 Fig). An informed consent was obtained from the owners of the animals prior to study enrollment. Procedures were approved by the local Ethic Committee of the University of Parma.

**Table 1:** Clinical cases.

Case number	Species	Age	Weight	Sex	Lesions
Case 1	*Testudo hermanni hermanni*	31 y	1200 gr	F	Multiple fractures of shell caused by a car 10 days earlier.
Case 2	*Testudo hermanni bodgeri*	24 y	1340 gr	M	Penetrating injury at the level of the distal humerus with exposed bone and loss of sensation in the terminal part of the limb 30 days earlier
Case 3	*Testudo hermanni*	unknown	650 gr	M	Serious injury of the radio ulna with loss of tissue and bone exposure by about 60 days
Case 4	*Testudo hermanni*	unknown	500gr	M	Serious injury with tissue loss and necrosis at the level of femur, by about 50 days

**Table 1 footnote**: Four turtles (Testudo spp.) with traumatic injuries were treated with TLRP. A description of the clinical outcome of the treatment is reported in Supporting Information Files S1 Fig, S2 Fig, S3 Fig, and S4 Fig.

### Statistical analysis

Fisher’s exact test was used to evaluate differences in the percentage of leukocytes and thrombocytes recovered from whole blood samples of *Testudo spp* and *Trachemys scripta elegans*. Statistical analysis was performed with WinPepi 10.2. Data are presented as means ± standard error of the mean (SEM). Fisher’s exact test was also used to compare the time needed to form a gel starting from cell-poor plasma or TLRP. Statistical significance was accepted at P<0.05.

## Results

### A high percentage of thrombocytes and leukocytes can be recovered by centrifugation of turtle’s blood samples

The method here proposed resulted in a better separation of leukocytes and thrombocytes on an Hystopaque layer when compared with the methods described in the literature for both *Testudo spp*. and *Trachemys scripta elegans* [19].

**Fig 3:**
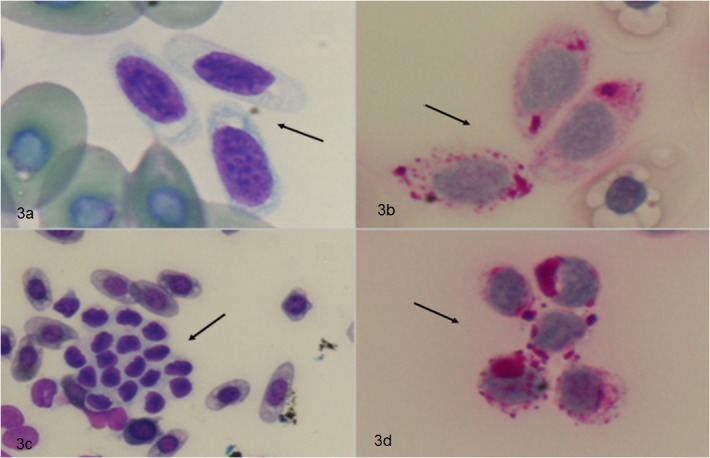
Upper row: Turtle whole blood cell staining with Diff Quick (a) and PAS (b). Lower row: TLRP smear stained with Diff Quick (c) and PAS stain (d). Thrombocytes are characterized by a round or oval shape, with a pale cytoplasm positive to PAS stain. (a),(b),(d): 100x magnification. (c): 40x magnification.

When centrifugation on Histopaque layer was perfomed at 150xg for 20 minutes, leukocytes and thrombocytes formed an evenly dispersed opaque cloud clearly separated from the erythrocyte pellet, as visible in Fig 2B (blue arrow). Quantitative analysis of the recovery of LL/TT (Table 2) resulted in 51.1% (± 4.9 SEM, n = 12) in testudo SSP and 46.3% (± 6.6 SEM, n = 10) in Trachemys Scripta Elegans. No statistically significant difference was observed between blood samples collected from different turtle species. In sum, considering the results obtained in the different species, the mean recovery of LL/TT was 48.9% (± 4.0 SEM, n = 22) of the whole blood cell content (Table 2). The examination of thrombocyte-leucocyte rich plasma (TLRP) smears confirmed the presence of thrombocytes and leukocytes in the preparation. In particular, thrombocytes appeared oval or round shaped, with a basophilic nucleus and a pale cytoplasm containing PAS positive vacuoles and granules (Fig 3) [20].

**Table 2:** Separation of leukocytes and thrombocytes on Hystopaque layer.

TESTUDO spp.	GENDER	RECOVERED LL/TT (%)	TRACHEMYS SCRIPTA ELEGANS	GENDER	RECOVEREDLL/TT (%)
	F	36.1 H		F	42.5
	M	38.3 H		M	20.0
	M	26.6 H		F	29.2
	F	44.6 H		F	36.8
	M	50.3 H		F	30.4
	M	44.6 H		M	57.3
	M	56.0 H		F	60.3
	M	48.5 H		F	93.8
	M	66.0 H		F	48.5
	F	72.7 Ma		F	44.0
	F	41.4 G			
	F	87.8 G			
**mean**		51.1	**mean**		46.3
**SEM**		±4.9	**SEM**		±6.6
Total		Animals	Mean		SEM
		22	48.9		±4.0

**Table 2 footnote**: Leukocytes and thrombocytes (LL/TT) recovery using a cell fractionation step on Hystopaque 1.077 at 150g x 20 min, RT in *Testudo spp*. (n = 12) *(T*. *Hermanni*, H; *T*.*Grecae*, G; *T*. *Marginata*, Ma) and *Trachemys scripta elegans* (n = 10). Values were not significantly different between the two groups (P = 0.4). Since the number of samples was extremely low for T.marginata and T. graeca, these species were excluded from the statistical analysis.

Instead, when the Histopaque gradient was centrifuged at 480xg for 30 minutes, as proposed by Morgan et al. [19], an opaque layer of white cells could be clearly separated at the interface between Histopaque and the phosphate saline buffered solution (Fig 2A, black arrow). Cell count demonstrated that a lower fraction of the total leukocytes and thrombocytes present in the unprocessed blood could be recovered from this cell layer. (9.9% ±1.1 SE, n = 3).

### TLRP jellifies

TLRP can be administered by dripping, spraying or injection or, alternatively, can be placed on the injured tissue as a gel that helps to spread and keep the TLRP on the desired location.

Jellification of the TLRP was obtained in glass test tubes and plates, by the addition of a 100 mg/ml calcium gluconate stock solution. A reliable and quick clotting of TRLP was obtained starting at 5% (v/v) stock solution at RT. The mean clotting time ranged between 8±2 and 14±4 min, for different calcium gluconate concentrations (Table 3). The gelation time of TLRP was significantly different from that obtained with cell-poor plasma in the same experimental conditions (P<0.05). No statistically significant difference was observed at 37°C. Furthermore, also TLRP lysate could be induced to gelation by adding stock solution of calcium gluconate higher than 5% v/v. Although the mean time of gelation was slightly lower at each calcium gluconate concentration tested, the results were not significantly different (data not shown).

**Table 3:** Cell-poor plasma and TLRP gelation.

Calcium gluconate (v/v)	2,5%	5%	10%	20%
**PLASMA**	No gelation	No gelation	20 (±6) min	20 (±4) min
**TLRP**	No gelation	14 (±4) min	10 (±4) min	8 (±2) min

**Table 3 footnote**: Time interval needed to obtain the gelation of pooled LL/TT poor plasma or TLRP after addition of 2.5 to 20% v/v of 100 mg/ml calcium-gluconate stock solution, in glass tubes. The time indicated is the mean time needed for the gelation of 4 different pooled samples, at room temperature (22°C). The gelation time of TLRP was significantly different from that obtained with cell-poor plasma (P<0.05) starting from 5% v/v calcium-gluconate stock solution.

### Clinical outcome following TLRP application is positive

**Fig 4:**
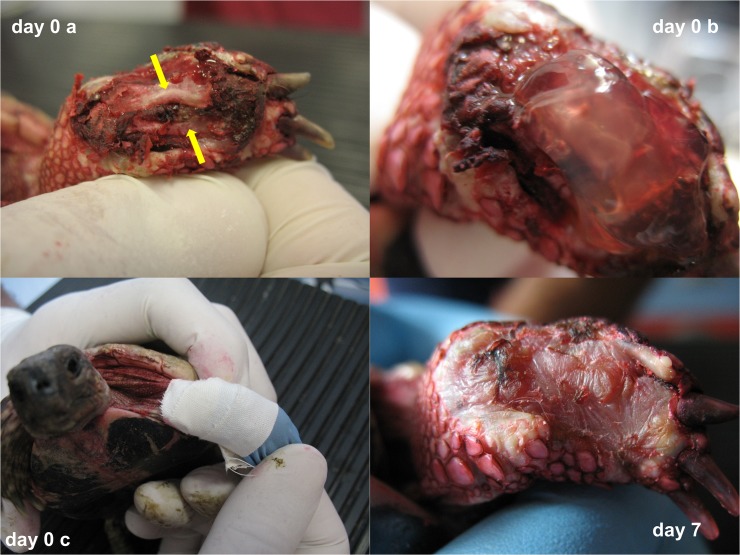
A severe traumatic injury with bone exposition (radius and ulna, yellow arrows), was referred 60 days after the accident. TLRP was applied as a gel. After application, the limb was bandaged. Seven days later the lesion was healed, with the production of a protective fibrotic tissue.

In order to have a preliminary evidence of the efficacy of TRLP in contributing to tissue healing, we chose injuries that were different in nature and extension. In two of them, the TLRP was injected, while it was applied as a gel in two other cases. All the patients healed and returned to their normal quality of life in a few weeks. An active and organized granulation tissue appeared shortly after the treatment (48 hrs) and the re-epithelisation of the exposed tissues was observed after a few days. Furthermore, the treatment induced the formation of a hardened tissue providing a mechanical protection against bacterial and/or mycotic contamination and tissue dehidration and dessication. Notably in one of the clinical cases described (Fig 4), the formation of a granulation tissue and the formation of a new tissue filling the wound defect was observed only following LTPR application after conventional therapies failed to induce tissue regeneration. No signs of bacterial contamination were observed after the application of TLRP. The outcome of the four clinical cases is described in Supporting Information Files S1 Fig, S2 Fig, S3 Fig, and S4 Fig.

## Discussion

The main objective of this work was the optimization of a protocol for the preparation of a thrombocyte concentrate to be used in regenerative medicine in turtles, as already done with platelet concentrates in mammals. Applying a two step gradient centrifugation protocol, we were able to recover about 50% of thrombocytes and leukocytes present in blood samples and to obtain an enriched plasma that has been used to treat traumatic injuries in four patients.

Platelet concentrates can be obtained in mammals by means of protocols [13, 21] that are based on differential centrifugation steps that allow cell fractionation exploiting the different sedimentation characteristics of nucleated versus non nucleated cells. The main problem for blood cell fractionation in reptiles is that erythrocytes, leukocytes and thrombocytes are all nucleated. Consequently, a single centrifugation step of the whole blood sample is not suitable to separate white blood cells from erythrocytes, as in mammals. The literature reports protocols for the separation of erythrocytes from other blood cells in turtles [19]. To the authors’ knowledge, however, no protocol has been developed with the aim of optimizing the quantitative recovery of the leukocyte/thrombocyte population. The method presented in this study is based on the use of Histopaque 1.077, a reagent widely used for blood cell fractionation in mammals, as well as in reptiles and birds. The recovery of white cells was optimized by comparing different methods for sample handling and centrifugation conditions, modifying the method proposed by Morgan et al. [19].

After blood sample collection, a first centrifugation step was applied to separate plasma from the blood cell fraction (Fig 1). The recovery and storage of the whole volume of the cell-poor plasma was necessary for the preparation of the TLRP in the final step of the procedure. The cell pellet, consisting of white as well as red blood cells, was resuspended in a volume of PBS equal to that of the removed plasma. The suspension was then loaded on a Histopaque layer. Cell separation on the Histopaque layer was impaired without this resuspension step, likely due to the high density of the cell suspension (data not shown).

The higher percentage of LL/TT recovery with respect to the blood sample total count, was obtained by centrifuging at 150xg for 20 minutes at room temperature. In these conditions, the white cell population was distributed between the ring visible within the Histopaque fraction and the interface between the Histopaque solution and the red blood cell fraction at the bottom of the tube (Fig 2B, blue arrow). Diff-Quick and PAS staining of cytological slides prepared from TLRP confirmed the presence of thrombocytes and leukocytes and demonstrated that these cells maintained their morphological characteristics (Fig 3) [20]. Centrifugation performed at higher gravitational fields or for longer times resulted in a lower cell recovery, although the cell layer inside the Histopaque solution appeared more distinct (Fig 2A, black arrow). Further molecular and morphological analyses are needed to identify the cell distribution in the two different regions of the gradient.

Since the aim of the protocol was to prepare a cell-enriched plasma suitable for clinical application in the field, experimental conditions capable of allowing gel formation from the TLRP in relatively short time (i.e. minutes) were evaluated at 22–24 °C (room temperature) and 37 °C. Both temperature values can be set in incubators and thermostatic baths usually available in normally equipped veterinary clinics and laboratories. The use of different types of substrates for the gelification process (i.e. plastic- and glass-ware) was also tested. The addition of 5–20% (v/v) of a 100 mg/ml solution of calcium gluconate, at both temperatures, gave a reliable coagulation in a time suitable for the clinical application (8–14 min), when performed in glass dishes or tubes as a support. Longer time was needed when plastic-ware was used and the resulting gel was less homogeneous and less stable.

Several approaches and strategies for the preparation of platelet concentrates have been described for mammals [11, 22, 23]. Although most of the preparations obtained are called PRP, the different experimental protocols applied lead to distinct products containing different cell populations [13]. Various parameters have been proposed to describe and define the characteristics of platelet concentrates suitable for clinical applications both in human and veterinary medicine. A first parameter is the efficiency of the method for platelet and leucocyte collection. The efficiency is considered “good” when cell recovery ranges between 40 and 80% of those present in the starting blood sample, while it is considered “excellent” for values higher than 80%. A second parameter proposed for the classification of platelet concentrates is the distribution of the different cell populations contained in the preparation. Pure PRP, in principle, should contain mostly platelets, while leucocyte-rich PRP (L-PRP) should contain, at variable ratios, both kinds of cells. Really pure PRP is considered difficult to prepare since platelets and leukocytes can sediment together during the centrifugation steps [24]. Although different protocols and commercial kits for the preparation of L-PRP are commonly applied in human and domestic animal clinics, the real cell composition is often undefined [25, 13]. A third parameter to be considered is the ratio between the volume of the processed blood sample and that of the platelet-rich plasma prepared. This ratio, as well as the efficiency of cell recovery from whole blood sample, has a marked effect on the final cell concentration and hence on the biological activity of the preparation. It is possible to collect and process large initial volumes of blood samples (10 to 50 ml or more) from humans and different species of animals (horse, large size dogs), thus obtaining several milliliters of PRP and/or L-PRP concentrates. These preparations usually give a 3- to 10- fold increase of platelet concentration compared to blood baseline values. Lower increments are obtained when blood is collected from small sized domestic animals, from which only a few milliliters (2–10) of blood can be drawn.

Clearly, each of these parameters must be considered if applying similar methods to the preparation of platelet concentrates in turtles. With regard to cell collection, our method allowed the recovery of approximately 50% of white blood cells (leukocytes and platelets), thus the collection efficiency can be defined as “good”. The preparation, as outlined by the acronym TLRP, contained both thrombocytes and leukocytes and thus it can be compared to mammalian L-PRP, where a net separation of the two cell populations is not achieved. Clear biological differences between pure PRP and L-PRP have not been documented in mammals either *in-vitro* or *in-vivo*, and both of them have been extensively used in clinical applications. Leukocytes contained in L-PRP could exert antimicrobial and/or immune regulation functions [26, 27] as well as contribute to tissue angiogenesis through VEGF production [28]. Some authors report a possible role of L-PRP in reducing pain and inflammation [29, 21]. Indeed, the respective effects of platelet and leukocytes on the biological activity of these concentrates have not yet been elucidated. Therefore, there is currently no basis for recommending that the elimination of leukocytes from the PRP might enhance its biological function in stimulating tissue regeneration [29].

The third parameter, i.e. the concentration of cells in mammalian PRP in correlation to its biological effects, has been extensively discussed. It has been reported that different protocols give variable platelet concentration increments [23]. PRP and L-PRP should have platelet concentration levels at least 3–5 times higher with respect to those of the unprocessed blood. On the other hand, some authors observed that higher concentrations could have negative effects on the in vitro activity of these preparations [30]. Furthermore, data concerning dose-dependent effects *in-vivo* are limited and little is known about the platelet concentration needed to obtain the most relevant therapeutic effect in clinical practice.

Sample collection in turtles is necessarily limited to a small amount of blood (1–3 ml) and, in the clinical practice the final concentration of platelets and leukocytes will depend on the volume of plasma needed for the application.

The *in vitro* and *in vivo* effects of platelet concentrates in mammals have been studied extensively. PRP on the one hand stimulates the replication of several cell types (fibroblast, mesenchymal stem cells, osteocytes, chondrocytes, etc.), and on the other, can modulate cell differentiation and angiogenesis. Furthermore, a significant antimicrobial action for platelet concentrates has been recently demonstrated [31, 32]. *In vivo*, application in orthopaedics (bone defects, arthropathies, tendon injuries), chronic ulcers and soft tissue regeneration have been reported, although with occasional contrasting results [33, 34]. This may be due to the lack of protocol standardization and to the still uncertain contribution of platelet degranulation to tissue healing [13].

The availability of a thrombocyte concentrate capable of stimulating tissue regeneration in turtles would be useful in improving the healing time of both soft tissue wounds and shell fractures. In this study, we have provided evidence that a plasma preparation enriched in thrombocytes and leukocytes can be obtained by means of a centrifugation protocol. Furthermore, the preparation can be induced to form a gel upon addition of calcium gluconate and thus can be applied on tissue lesions both as a liquid or a gel, depending on clinical requirement.

To evaluate a possible contribution of thrombocyte concentrates to both soft tissues and shell healing in chelonians, we have treated traumatic injuries in four different clinical cases. Although our data are relative to a low number of cases, the clinical outcome suggests that TLRP therapy is safe and could contribute to both soft tissue and shell healing. As a matter of fact, the rapid re-epithelisation and the formation of a hardened tissue avoided the contamination of the exposed tissues and protected the healing tissue from dehydration and desiccation also in non-healing chronic wounds that were previously treated with conventional therapies. Given the low number of clinical cases treated, the heterogeneity of the lesions and the lack of control animals, definitive conclusions cannot be drawn about the efficacy of the LTRP treatment in comparison to conventional therapies. Nevertheless, our data suggest that the use of thrombocyte-derived products could represent an alternative or a support of traditional therapies towards which they do not have any incompatibility or contraindication. Further studies will be necessary to optimize the protocol for the preparation of LTRP as well as to achieve a better cellular and molecular characterization of the preparation and its contribution to tissue healing.

## Supporting Information

S1 Fig Case 1. Multiple fractures of shell caused by a car 10 days earlier. All fractures were immobilized with external fixators. Forty-eight hours after surgery, an abnormal hyperaemic reaction was noted involving the ventral side of the left bridge (pectoral and abdominal scuti). The entire affected area was mobile and there was little adherence to the surrounding tissues. The use of screws and cerclage did not prove to be effective in immobilizing the area, so the authors decided to infiltrate TLRP in liquid form below this area in order to facilitate the healing of scuti (Figure, day 1). At day 15 post-inoculation, a line of white tissue surrounding the wound edges was visible (Figure, day 15, yellow arrow). A sample of this white tissue was collected for histological examination, which showed the presence of keratin lamellae (fig case 1, histology). Three months later, the healing process was complete and the injured scuti were adherent to surrounding tissues and completely ossified, requiring no further treatment. The remaining lesions of the shell have required an additional three months to complete healing (Figure, day 180).
(TIF)

S2 Fig Case 2. Penetrating injury at the level of the distal humerus with exposed bone and loss of sensation in the terminal part of the limb, 30 days earlier. The amputation of the distal portion of the limb at the level of the elbow joint was performed. Because the skin available was not sufficient to cover the entire articular surface of the humerus, after 30 days the lesion was not healed and we opted for the use of TLRP. After application of the TLRP gel, the limb was bandaged. Ten days after the first application, the ulcer was completely healed and a thick fibrous callus had formed on the humerus, which allowed the patient to move autonomously inside the terrarium (figure, day 18).
(TIF)

S3 Fig Case 3. Injury of the radius and ulna, with loss of tissue and bone exposure for about 60 days. A severe traumatic injury with bone exposition (yellow arrows) was referred 60 days after the accident. The lesion was medicated and necrotic tissue was removed. We opted to use TLRP as a gel. After application, the limb was bandaged. No other therapy was performed. After 24 h, the lesion appeared markedly improved and 24 h later fibrotic tissue completely covered the two exposed bones. Seven days later the lesion was completely healed.
(TIF)

S4 Fig Case 4. Serious injury with tissue loss and necrosis at the level of femur, by about 50 days. The animal was referred with a severe traumatic skin lesion at the level of the femur. The skin was sutured (figure, day 0). After fifty days, the tissue appeared necrotized(day 0, right). All the necrotic tissue was removed and TLRP treatment was performed. After 24 h the lesion appeared improved and a second application was performed (figure, day 1). After 5 days the necrotic tissue had completely disappeared and a thick fibrous callus had formed (figure, day 5).
(TIF)

S5 Fig Step by step summary of TLRP preparation. **Step 1.** The blood sample is collected from the caudal or cervical vein and centrifuged at 250xg for 15 min. Acid Citrate Dextrose is used as anticoagulant. **Step 2.** The plasma fraction (cell-poor plasma, devoid of cells) is transferred to a sterile tube and stored to be used in step 4. **Step 3.** The cell pellet is resuspended in a volume of physiological saline equal to the volume of cell-poor plasma collected. Then cell suspension is loaded on Histopaque and centrifuged at 150xg for 20 min. Red blood cells are separated from thrombocytes and leukocytes. **Step 4.** The thrombocyte/leucocyte fraction is collected, washed twice in 10 ml of physiological saline and counted. Finally, cells are resuspended in a suitable volume of cell-poor plasma to obtain the TLRP. The volume of cell poor plasma is determined as a function of the desired cell concentration (3x-5x) and the area of the lesion to be treated with the TLRP. **Step 5.** TLRP can be applied as a cell suspension (by dripping, spraying, injection), as a gel, or as cell lysate.
(TIF)
