# Supplementary figures and images for: Preparation and Application of an Innovative Thrombocyte/Leukocyte-Enriched Plasma to Promote Tissue Repair in Chelonians
Source: PLoS One. 2015 Apr 22;10(4):e0122595. doi: 10.1371/journal.pone.0122595 (PMC4406685; doi:10.1371/journal.pone.0122595)

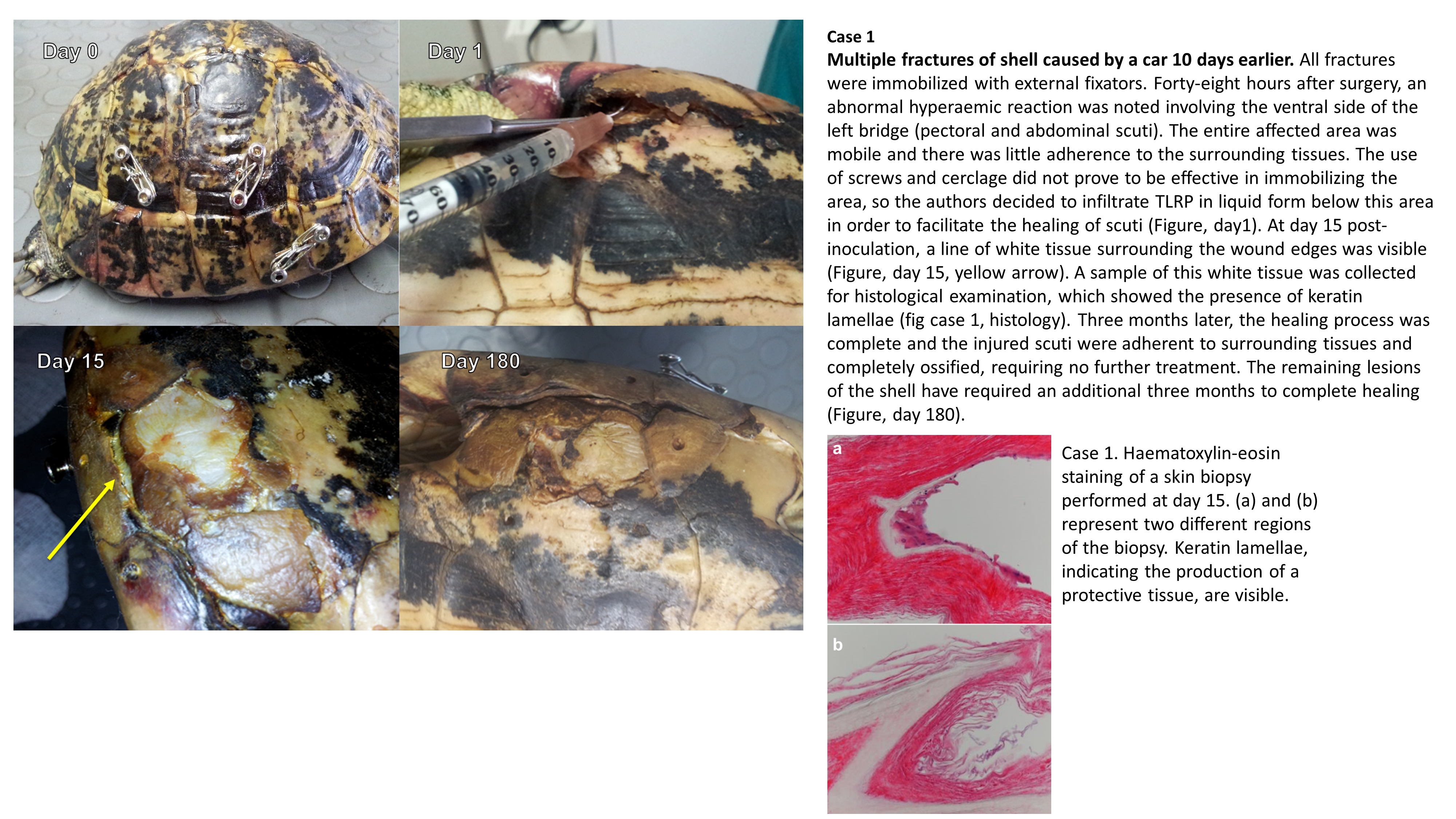

Supplement: S1 Fig — Multiple fractures of shell caused by a car 10 days earlier. All fractures were immobilized with external fixators. Forty-eight hours after surgery, an abnormal hyperaemic reaction was noted involving the ventral side of the left bridge (pectoral and abdominal scuti). The entire affected area was mobile and there was little adherence to the surrounding tissues. The use of screws and cerclage did not prove to be effective in immobilizing the area, so the authors decided to infiltrate TLRP in liquid form below this area in order to facilitate the healing of scuti (Figure, day 1). At day 15 post-inoculation, a line of white tissue surrounding the wound edges was visible (Figure, day 15, yellow arrow). A sample of this white tissue was collected for histological examination, which showed the presence of keratin lamellae (fig case 1, histology). Three months later, the healing process was complete and the injured scuti were adherent to surrounding tissues and completely ossified, requiring no further treatment. The remaining lesions of the shell have required an additional three months to complete healing (Figure, day 180). (TIF) [file pone.0122595.s001.TIF]

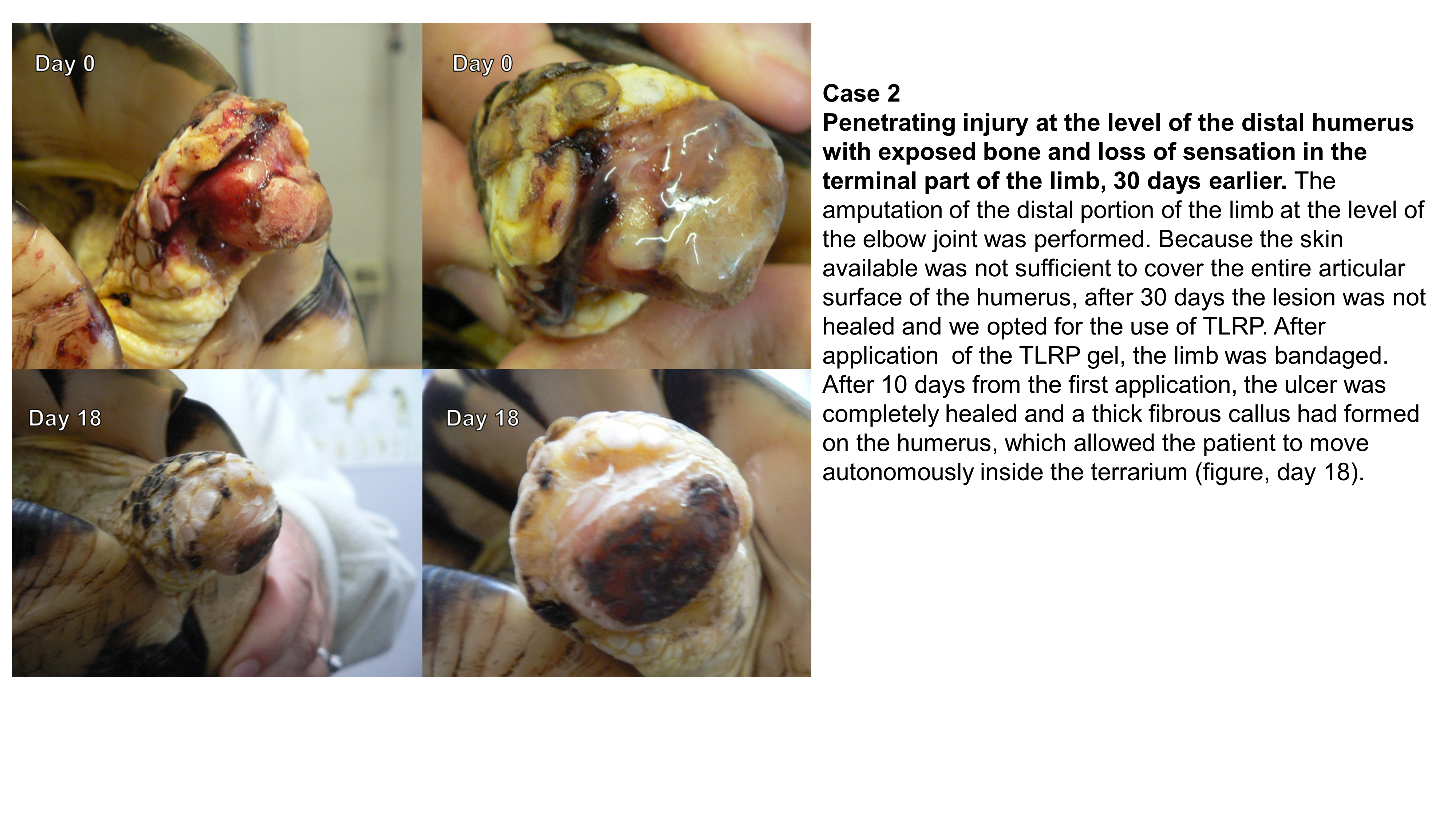

Supplement: S2 Fig — Penetrating injury at the level of the distal humerus with exposed bone and loss of sensation in the terminal part of the limb, 30 days earlier. The amputation of the distal portion of the limb at the level of the elbow joint was performed. Because the skin available was not sufficient to cover the entire articular surface of the humerus, after 30 days the lesion was not healed and we opted for the use of TLRP. After application of the TLRP gel, the limb was bandaged. Ten days after the first application, the ulcer was completely healed and a thick fibrous callus had formed on the humerus, which allowed the patient to move autonomously inside the terrarium (figure, day 18). (TIF) [file pone.0122595.s002.TIF]

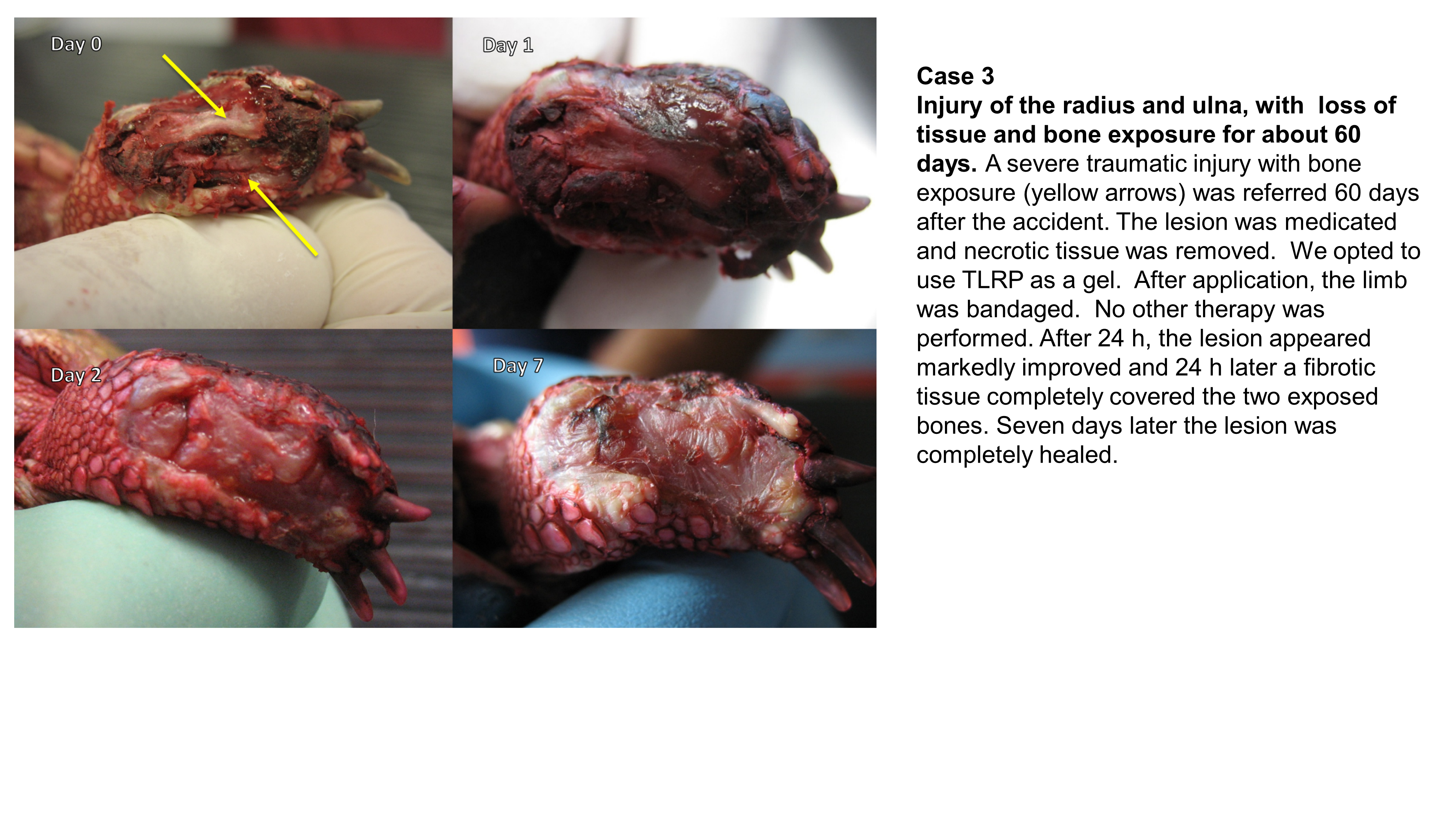

Supplement: S3 Fig — Injury of the radius and ulna, with loss of tissue and bone exposure for about 60 days. A severe traumatic injury with bone exposition (yellow arrows) was referred 60 days after the accident. The lesion was medicated and necrotic tissue was removed. We opted to use TLRP as a gel. After application, the limb was bandaged. No other therapy was performed. After 24 h, the lesion appeared markedly improved and 24 h later fibrotic tissue completely covered the two exposed bones. Seven days later the lesion was completely healed. (TIF) [file pone.0122595.s003.TIF]

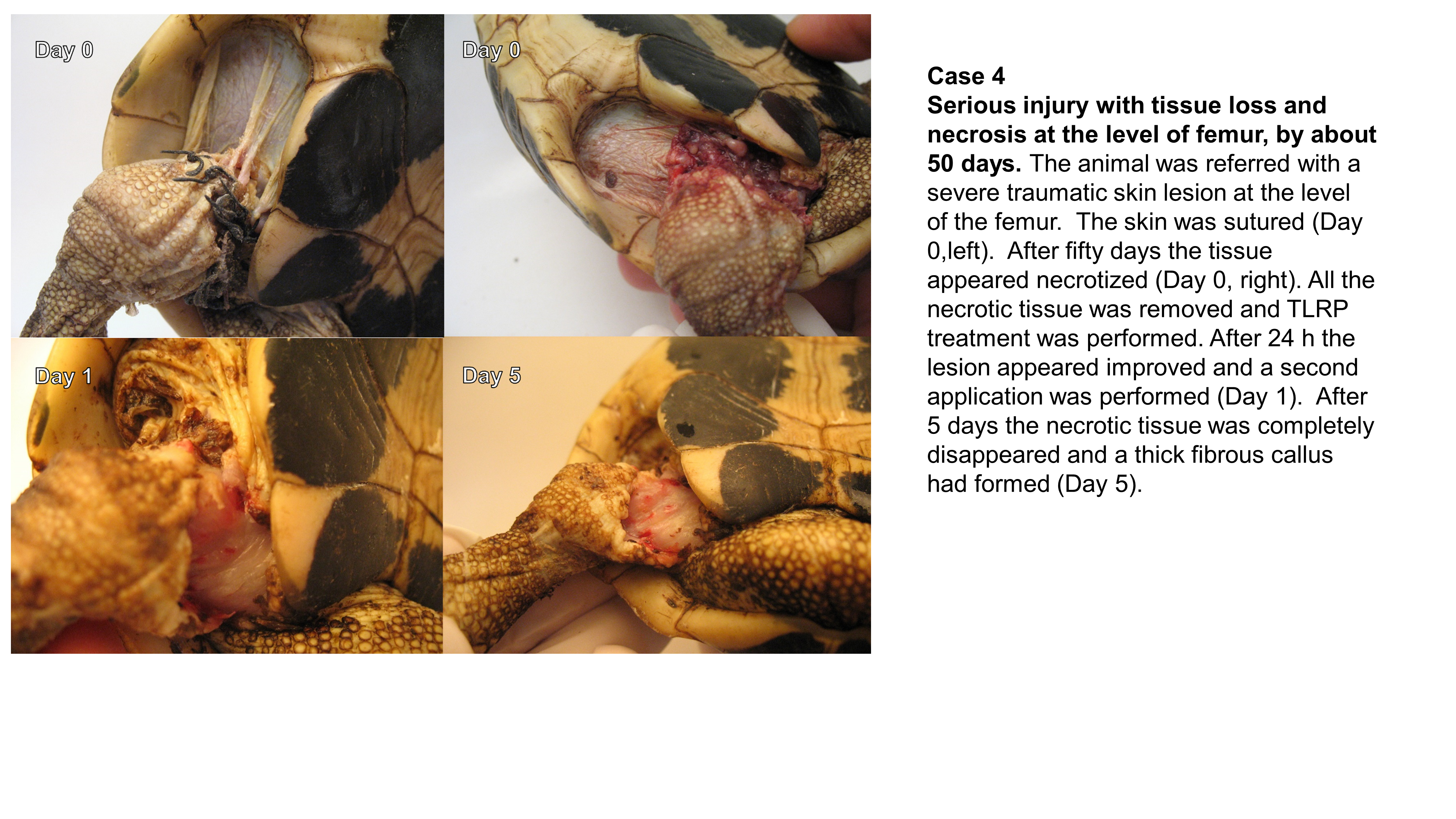

Supplement: S4 Fig — Serious injury with tissue loss and necrosis at the level of femur, by about 50 days. The animal was referred with a severe traumatic skin lesion at the level of the femur. The skin was sutured (figure, day 0). After fifty days, the tissue appeared necrotized(day 0, right). All the necrotic tissue was removed and TLRP treatment was performed. After 24 h the lesion appeared improved and a second application was performed (figure, day 1). After 5 days the necrotic tissue had completely disappeared and a thick fibrous callus had formed (figure, day 5). (TIF) [file pone.0122595.s004.TIF]

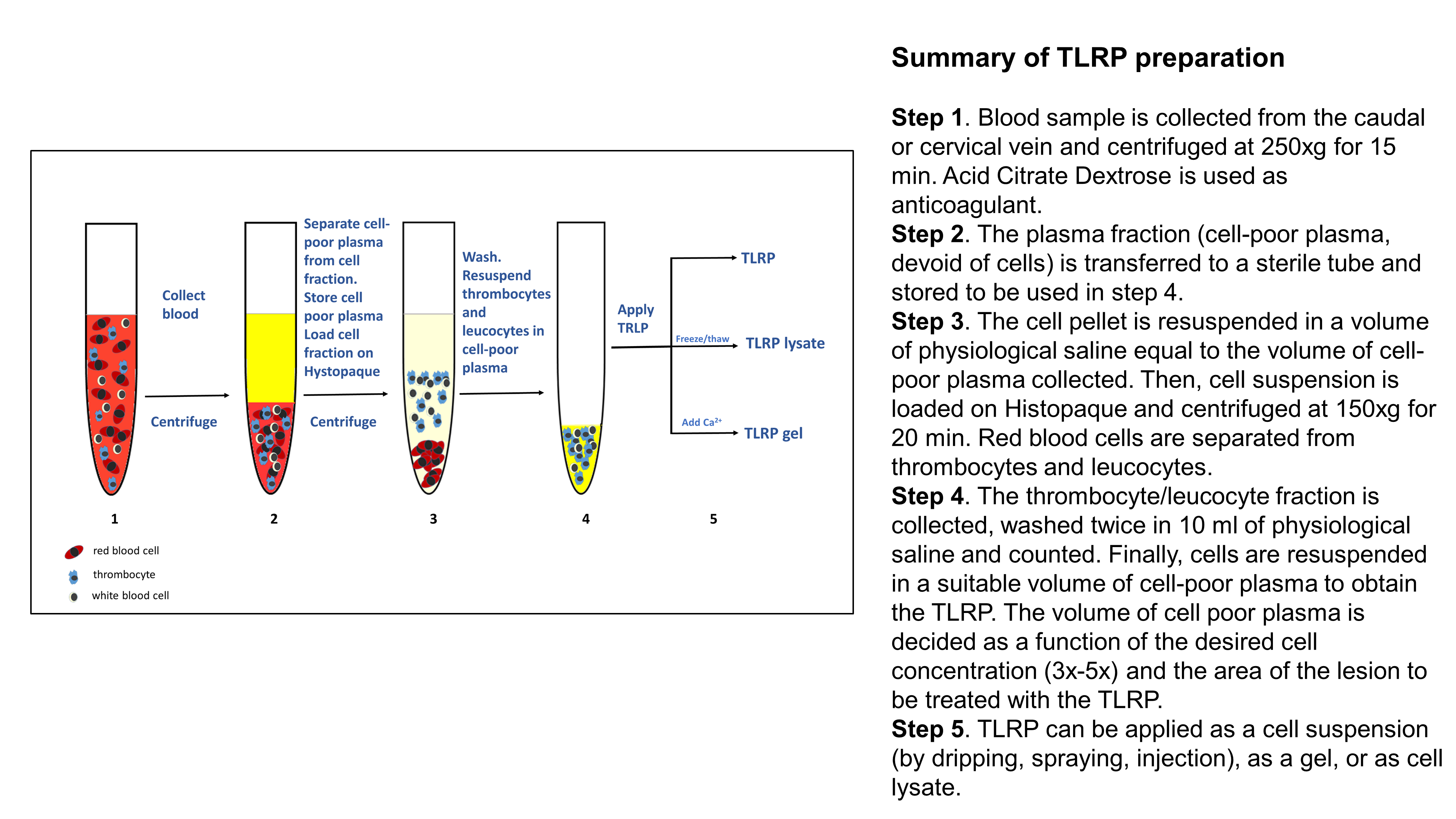

Supplement: S5 Fig — Step 1. The blood sample is collected from the caudal or cervical vein and centrifuged at 250xg for 15 min. Acid Citrate Dextrose is used as anticoagulant. Step 2. The plasma fraction (cell-poor plasma, devoid of cells) is transferred to a sterile tube and stored to be used in step 4. Step 3. The cell pellet is resuspended in a volume of physiological saline equal to the volume of cell-poor plasma collected. Then cell suspension is loaded on Histopaque and centrifuged at 150xg for 20 min. Red blood cells are separated from thrombocytes and leukocytes. Step 4. The thrombocyte/leucocyte fraction is collected, washed twice in 10 ml of physiological saline and counted. Finally, cells are resuspended in a suitable volume of cell-poor plasma to obtain the TLRP. The volume of cell poor plasma is determined as a function of the desired cell concentration (3x-5x) and the area of the lesion to be treated with the TLRP. Step 5. TLRP can be applied as a cell suspension (by dripping, spraying, injection), as a gel, or as cell lysate. (TIF) [file pone.0122595.s005.tif]
